# Expert-Augmented Computational Drug Repurposing Identified Baricitinib as a Treatment for COVID-19

**DOI:** 10.3389/fphar.2021.709856

**Published:** 2021-07-28

**Authors:** Daniel P. Smith, Olly Oechsle, Michael J. Rawling, Ed Savory, Alix M.B. Lacoste, Peter John Richardson

**Affiliations:** ^1^ BenevolentAI, London, United Kingdom; ^2^ BenevolentAI, Brooklyn, NY, United States

**Keywords:** drug repurposing, knowledge graph, human computer interaction, SARS-CoV-2, COVID-19, knowledge discovery and data mining

## Abstract

The onset of the 2019 Severe acute respiratory syndrome coronavirus 2 (SARS-CoV-2) pandemic necessitated the identification of approved drugs to treat the disease, before the development, approval and widespread administration of suitable vaccines. To identify such a drug, we used a visual analytics workflow where computational tools applied over an AI-enhanced biomedical knowledge graph were combined with human expertise. The workflow comprised rapid augmentation of knowledge graph information from recent literature using machine learning (ML) based extraction, with human-guided iterative queries of the graph. Using this workflow, we identified the rheumatoid arthritis drug baricitinib as both an antiviral and anti-inflammatory therapy. The effectiveness of baricitinib was substantiated by the recent publication of the data from the ACTT-2 randomised Phase 3 trial, followed by emergency approval for use by the FDA, and a report from the CoV-BARRIER trial confirming significant reductions in mortality with baricitinib compared to standard of care. Such methods that iteratively combine computational tools with human expertise hold promise for the identification of treatments for rare and neglected diseases and, beyond drug repurposing, in areas of biological research where relevant data may be lacking or hidden in the mass of available biomedical literature.

## Introduction

In late 2019 and early 2020 the new coronavirus SARS-CoV-2 spread rapidly from China to the rest of the world, with over 3 million deaths recorded as of April 2021. There was little time to identify effective therapeutics and certainly no time to develop new therapeutics and have them authorised by the regulators. Although little was known about the virus SARS-CoV-2 and its resultant disease COVID-19 at the turn of 2020, the experience of the SARS epidemic in 2003 offered some insights as to the characteristics of a suitable treatment. Both viruses resulted in protracted respiratory illness over several weeks, with resulting Acute Respiratory Distress Syndrome (ARDS), a major cause of death. It was thought (Xu et al., 2020; Zhao et al., 2020) that, as for SARS, the cell-surface protein Angiotensin Converting Enzyme 2 (ACE-2) could serve as a receptor for the new virus.

Recently, computer-enhanced methods for repurposing approved drugs have been developed (Zhou et al., 2020a; Jarada et al., 2020). These include AI approaches (Nadeau et al., 2020; Zhou et al., 2020b), and unbiased laboratory methods to identify the protein-protein interaction (PPI) networks operating between the virus and the host (Gordon et al., 2020). In another approach, Zeng et al. (2020) compiled a knowledge graph integrating scientific literature and drug properties in order to identify 41 approved drugs which could be used to treat COVID-19. Of these, the anti-inflammatory steroids have shown some efficacy (e.g. RECOVERY trial, Horby et al., 2021), hydroxychloroquine has generally not, and the remainder have not been tested in randomised clinical trials in this disease to our knowledge. The antiviral drugs that were available had been developed for the treatment of other viruses (e.g. lopinavir/ritonavir for HIV and remdesivir for Ebola), so there was a distinct possibility that they would not be effective against SARS-CoV-2.

We describe here how, in early 2020, we used a visual analytics approach using interactive computational tools in multiple iterations to identify promising potential treatments. We used BenevolentAI’s drug discovery knowledge graph (KG) of biomedical data (Paliwal et al., 2020) which comprises contents from dozens of biomedical databases, enhanced by information from machine-read scientific literature, covering the enormous amount of biomedical knowledge available (approximately 30 M papers are cataloged in PubMed). Although built for de-novo drug discovery, the inclusion of approved drugs, treatments and other information made it a suitable resource for drug repurposing. The KG includes information on drugs, genes and proteins alongside representations of mechanisms, processes and pathways. This representation of biology was a key enabler to specifically target host mechanisms subverted by the virus which could be safely targeted.

We searched for approved drugs with anti-inflammatory activity, to counter the overactive immune response that characterises severe COVID-19 (Huang et al., 2020), along with evidence of hitherto-unexplored antiviral properties. We describe here the identification of the mechanisms and pathways exploited by the virus in its infection of human cells, and the subsequent identification of putative drug targets and drugs. The outcome of the process was the identification of baricitinib, at the time approved as a treatment for Rheumatoid Arthritis, as a compelling candidate for treating patients with COVID-19.

## Materials and Methods

### Knowledge Graph Construction

The BenevolentAI KG is a compendium of numerous data sources represented as nodes and connecting relationships in a graph structure, designed specifically for use in de-novo drug discovery. Graph nodes are chiefly biomedical entities, such as diseases, disease processes, pathways, proteins and compounds, with directed relationships scoring various types of connection or association between entities. The KG includes data from both curated databases and literature sources, with its construction described in Paliwal et al. (2020). Importantly, the graph is enhanced for disease processes, mechanisms and pathways, which allow exploration of novel diseases through an understanding of the underlying biology.

Information extraction by Natural Language Processing (NLP) algorithms cover gaps in established curated data. This allows updates to the KG from the scientific literature on a regular basis resulting in nearly 20% of the KG relationships being derived from literature alone. The NLP pipeline that produces these relationships allows for the quick inclusion of new data permitting rapid augmentation of novel concepts, such as those pertaining to COVID-19 and SARS-CoV-2. The relationships between entities in the KG reflect the rich complexity of biomedical information including causal relationships, pathways, processes, group memberships, ontologies and hierarchies.

A knowledge graph representation of biomedical information (both curated and literature-derived) provides a unified and common structure for retrieving information. This facilitates the development and use of algorithms and computational tools capable of carrying out knowledge discovery and data mining (KDD) (Fayyad et al., 1996) within a drug discovery context. The particular workflow described here consisted of a combination of KDD and HCI methods (HCI-KDD) described by Holzinger (2013); the KDD methods of automated data mining and modeling of large data repositories, and the HCI methods of expert-driven, interactive and visual analysis; providing an effective iterative human-machine partnership that led to the identification of baricitinib as a potential treatment for COVID-19.

### Analysis Tools

Two interactive exploration tools were used during the initial stages of our COVID-19 research, a graph pattern querying tool and a protein-protein interaction (PPI) network analysis tool.

#### Graph Pattern Querying

The graph pattern querying tool is used for purposeful querying of the knowledge graph when the user has a specific question (e.g. is mechanism/pathway X involved in disease Y?) and, as in this case, to retrieve drug candidates which target specific mechanisms. Users sketch complex questions in the form of visual graph patterns consisting of known or unknown information, requiring the system to satisfy gaps using matching concepts, relationships and properties present in the knowledge graph.

To enable non-technical domain experts to successfully interrogate a large biomedical knowledge graph, an interactive and visual pattern-building interface provides an intuitive means for users to encode their questions and thoughts fluidly, demonstrated by Blau et al. (2002), Chau et al. (2008) and Pienta et al. (2016). The visual pattern being created also provides a common language between experts from different backgrounds - particularly biology and technology - resulting in a collaborative environment that helps fuse drug discovery expertise with data modeling expertise, to maximise retrieval of the most promising results.

**FIGURE 1:**
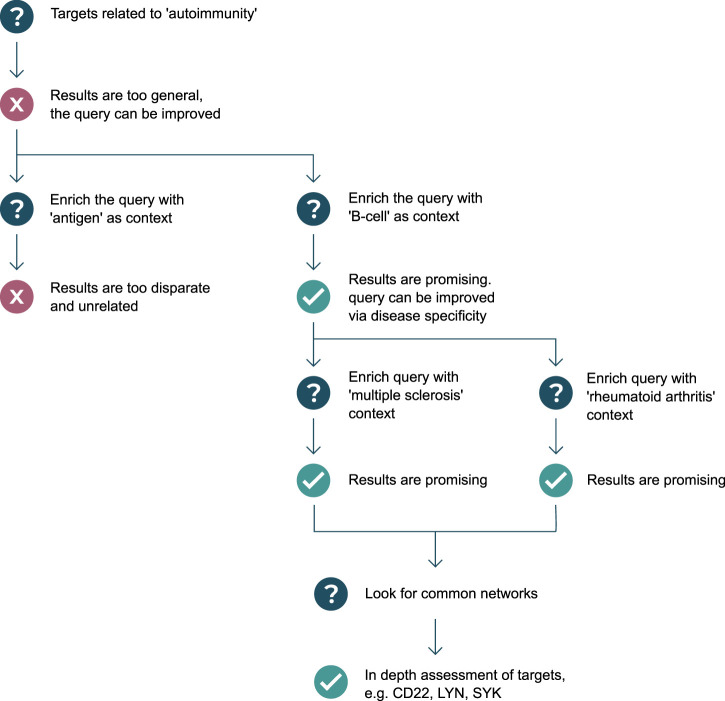
Example use-case of graph pattern querying: in search of targets regulating autoantibody production. Question mark symbols represent stages of asking questions of the knowledge graph, which can result in undesirable results or a failed attempt at querying the knowledge graph (represented by red cross symbols), or desirable results and successful attempts at querying the knowledge graph (represented by green check mark symbols). A diverging path represents the user exploring possibilities down both routes, either as a result of a failed attempt, or as a result of there being two equally valuable options to pursue. A converging path represents the user linking the results of two patterns together, in a new pattern.

In almost all scenarios, the question or graph pattern that is being built, is a product of multiple iterations of asking and elaborating on an initial question. Often, but not always, starting broad, and progressively getting narrower and more specific, the process of graph pattern querying is a dialogue between an expert user and the system, where the user explores what exists, what does not exist, and what is possible given the graph data model. Executing patterns incrementally helps users to assess whether graph pattern results are leading to relevant biological directions, and whether or not to backtrack or change strategy–resulting in a tree-like (or road map) journey of pattern creation, illustrated in Figure 1. The flexibility and complexity that are inherent in the construction of graph patterns are often representative of the complexity of biology itself, as well as the informational complexity required to model biomedical data in a graph structure, and for this reason, iterative pattern creation with fast feedback loops are important success factors in this workflow.

#### Network Analysis

The protein-protein interaction (PPI) network analysis tool is designed to make sense of genes, proteins and their interactions, and the emergent pathways involved in particular biological mechanisms. The network analysis carried out using this tool led to the identification of host pathways and processes likely to be subverted by the virus. The tool permits user manipulation of high-level biomedical concepts, such as diseases, biological processes and biological pathways, in the form of their protein-based representations and their interactions, which result in PPI networks.

This approach, in combination with a heterogenous knowledge graph data source, has been demonstrated to provide an effective means of analysing and reasoning over disease pathology at a high level (Goh et al., 2007; Lysenko et al., 2016). A useful feature set surrounds the network visualisation, which supports flexible importing of data from the knowledge graph, manual importing of data, as well as real-time importing from a literature database, enabling rapid exploration and sensemaking of different biological narratives, and importantly the assessment of hypotheses. The tool allows users to import gene sets from a number of sources, visualise them as networks via their PPIs, view and explore the biological relationships in those networks, and identify targets of therapeutic interest for a given indication. Representing diseases and biomedical concepts behind the pathology of diseases using gene sets is a fundamental approach to retrieving information from the knowledge graph, this is most commonly encountered in bioinformatics via gene set enrichment analysis (Subramanian et al., 2005), which provides higher-level conceptual summaries of gene sets using biological pathways, biological processes and other insightful conceptual terms. Upon importing gene sets, networks are automatically constructed using PPIs from a number of databases (Biogrid, STRING, KEGG, SiGNoR, OmniPath) that exist within the knowledge graph, and are spatially arranged using a force-directed layout algorithm (Fruchterman and Reingold, 1991). The accuracy and validity of PPIs exists on a spectrum—from computationally inferred to human curated. PPIs also exist on a spectrum of causality—from a non-directed, binary interaction, to a directed interaction of a particular mechanism (e.g. phosphorylation) and effect (e.g. activates). When retrieving PPIs from the knowledge graph, it is possible to set criteria to navigate these spectra in the tool, primarily to increase quality and clarity of PPIs within networks–an example might be to increase quality and causality if the network is overwhelmingly connected, or to reduce quality and causality if the network has many unconnected proteins. The criteria used in this workflow was that all PPIs must have a regulatory effect - either up-regulating or being up-regulated, or down-regulating/being down-regulated by their interacting neighbor.

**FIGURE 2:**
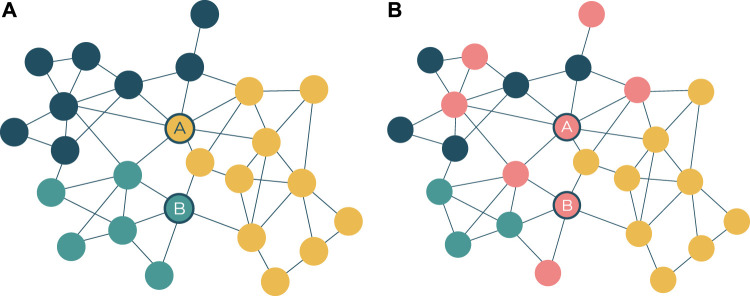
**(A)** Pivotal proteins (represented by nodes A and B) are loosely defined as proteins that facilitate cross-talk between network modules. This has some overlap with the notion of a node with high betweenness centrality (Freeman 1977), but there is emphasis on the node’s connectivity across network modules. Node A represents a target belonging to the yellow network module and interacts with the highest number of targets in the blue module. Node B represents a target belonging to the green network module and interacts with the highest number of targets in the yellow module. **(B)** Pathway membership, represented by nodes colored red, can be scattered across different network modules. While the modules in the network may represent distinct GO processes, biological pathways serve multiple such processes and are therefore seldom confined to one module.

Complementary to the visual network structure that emerges from the force-directed layout algorithm, topological communities are also automatically identified and visualised to provide rough topological modules of potential mechanistic roles. The community detection algorithm used in this tool, and this research, is the Louvain algorithm (Blondel et al., 2008), which leads other community detection algorithms in terms of network size and computational efficiency (Rahiminejad et al., 2019). The outputs of community detection on these networks result in a visual and interactive set of network modules that are often, but not always, functionally distinct from one another. The advantages inherent in such a visualised network structure include the identification of pivotal proteins connecting different parts of the network (Figure 2A), as well as the roles of specific pathways and processes in and between the topological clusters (Figure 2B).

Further biological contextualisation of the network is supported through mining literature where the top proteins associated in paragraphs with a given text-based query, can be imported into the network instantaneously, or if existing in the network already, characterised based on their relevance to the search phrase. “Top” is determined by two factors–the raw count of paragraphs in which they co-occur (i.e. strength of association), and a normalised point-wise mutual information score (Bouma 2009; Watford et al., 2018) that captures how they occur independently of each other as well as together (i.e. specificity of association). For biological concepts that are most accurately described with nuanced phrases, and for mitigating a lack of data coverage for particular biomedical entities, phrase searching provides a flexible input (text-based, multi-part, word inflections) and a high recall (paragraph-level granularity). These combine to provide an effective literature extraction technique for phrase-protein relationship extraction. In this way, the user can explore and understand disease processes through PPIs, pathways, biological processes, mechanisms and diseases simultaneously, continuously adding and removing additional nuanced biological concepts to test assumptions and follow their train of thought fluidly, significantly enriching the exploration.

In this workflow, we focused on mechanisms related to viral infection and the host inflammatory response to the virus, via the creation and analysis of four networks illustrating the proteins, pathways and processes which underlie these mechanisms.

### Statistical Tests

Fisher’s exact test was used to determine the relevance of enriched pathways and processes in specific gene sets. The SciPy implementation in python, together with a contingency table was used. The formula takes into account 3 variables: genes in the network, genes in the pathway, and all genes in the KG. The *p*-value is computed as follows:

$$p\;=\left(\frac{\left(a+b\right)}{a}\right)\;*\frac{\left(\frac{\left(c+d\right)}{c}\right)\;}{\;\left(\frac{N}{\left(a+c\right)}\right)}$$

with the values as represented in the below contingency Table 1.

**TABLE 1:** Top 10 host proteins, pathways and GO processes associated with SARS-CoV-2 infection.

Rank	Protein	Protein relationships	Pathway	Pathway relationships	Process	Process relationships
1	ACE2	6,413	Immune system	12,237	Positive regulation of transcription by RNA polymerase II	5,287
2	CDSN	3,873	Cytokine signaling in immune system	9,004	Viral entry into host cell	4,513
3	TMPRSS2	1,279	Metabolism of proteins	8,201	Signal transduction	3,815
4	DPP4	1,132	Signal transduction	7,345	Positive regulation of gene expression	3,490
5	ANPEP	1,032	Disease	4,109	Positive regulation of cell population proliferation	3,212
6	CD8A	816	Gene expression (transcription)	3,095	Negative regulation of transcription by RNA polymerase II	3,150
7	IFNG	774	RNA polymerase II transcription	2,960	Positive regulation of transcription, DNA-templated	3,141
8	CLEC4M	747	Post-translational protein modification	2,945	Negative regulation of cell population proliferation	2,895
9	CTSL	742	Generic transcription pathway	2,940	Positive regulation of NF-kappaB transcription factor activity	2,390
10	IL6	726	Signaling by receptor tyrosine kinases	2,802	MAPK cascade	2,361


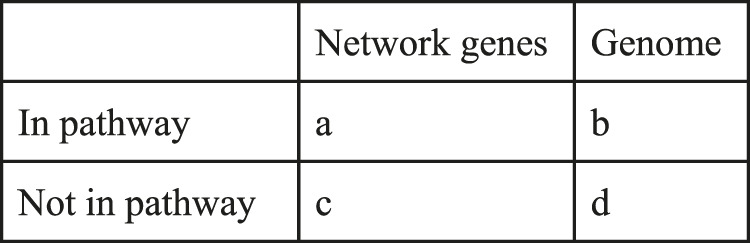


The *p*-value is adjusted for the false discovery rate using the Benjamini and Hochberg procedure (Benjamini, 2010).

## Results

COVID-19 is a novel viral disease, and the graph did not originally include significant amounts of pertinent information as of January 2020. We therefore first augmented the graph with coronavirus related information from recent literature through our NLP pipeline. This brought approximately 40,000 additional relationships into the graph. New relationships consisted of disease, biological process, tissue and compound entities, related together through an unsupervised, rule-based model at the sentence level (“SVO,” see Paliwal et al., 2020).

**FIGURE 3:**
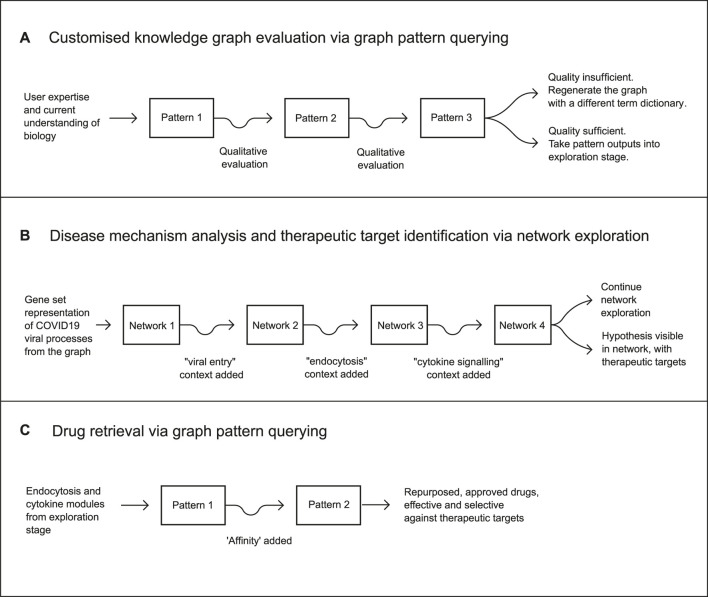
The full drug repurposing workflow consisted of three stages. The first stage incorporated the evaluation of a customised knowledge graph via graph pattern querying as a means of rapid qualitative evaluation. The second was a mechanistic analysis of viral and host processes, via the creation of an initial network using a gene set representation of COVID19-related processes extracted from the customised knowledge graph. This was iterated three more times to achieve a final network containing a clear hypothesis and therapeutic targets. The third stage resulted in a hypothesis as a partially defined graph pattern, from which targets and drug candidates were retrieved, producing a final set of candidate treatments for COVID-19.

The repurposing workflow was then executed across three stages: a graph pattern evaluation workflow for assessing the quality of the customised knowledge graph, followed by a network analytics workflow leading to the identification of therapeutic mechanisms and druggable targets across those mechanisms, and a final graph pattern querying workflow identifying a number of approved drugs suitable for inhibiting the target biology (Figure 3).

### Evaluation of the Knowledge Graph with Customised SARS-CoV-2 Relationships

Using the graph pattern querying tool, we performed a qualitative assessment of the customised KG, which we had enriched with coronavirus related material. Three different queries were used to identify the host proteins, pathways and processes impacted by the virus. Table 2 lists the top ten host proteins, host pathways and host biological processes identified as being closely related to SARS-CoV-2 biology, identified using a relational graph pattern query, created using the graph pattern query tool. They are ranked according to the number of graph relationships with the SARS-CoV-2 biology. The top ten host proteins included the coronavirus receptors ACE2 and DPP4, the protease TMPRSS2 (which is highly implicated in the activation of the SARS-CoV-2 spike protein), and inflammatory cytokines most of which were already implicated in COVID-19 disease. In the top ten pathways implicated were angiotensin metabolism, interferon alpha/beta signaling and cytokine signaling. The top host biological processes associated with the virus included viral entry into the cell and a host of inflammatory processes. This exercise confirmed that the enriched graph contained proteins, pathways and processes relevant to coronavirus infection. After these three iterations of pattern querying, a protein set representation of the SARS-/host interactome (556 proteins) from the customised graph was used to create the first network.

**TABLE 2:** Top 20 enriched biological pathways (Reactome) in the final network.

Rank	Name	*p* value	Matched	Gene set
1	Immune system	1.32E-102	343	2,129
2	Cytokine signaling in immune system	6.36E-82	222	875
3	Signaling by interleukins	2.02E-66	155	458
4	Clathrin-mediated endocytosis	3.47E-40	75	145
5	Innate immune system	1.65E-27	150	1,044
6	Interleukin-10 signaling	3.91E-25	37	45
7	Cargo recognition for clathrin-mediated endocytosis	8.56E-25	49	105
8	Toll-like receptor cascades	3.97E-24	56	153
9	Vesicle-mediated transport	3.10E-23	111	669
10	Membrane trafficking	1.83E-21	104	630
11	Interleukin-4 and Interleukin-13 signaling	1.58E-20	44	108
12	Toll like receptor 4 (TLR4) cascade	8.76E-20	46	127
13	Interleukin-2 family signaling	3.31E-17	28	44
14	Interferon alpha/beta signaling	4.17E-17	33	70
15	Chemokine receptors bind chemokines	1.19E-16	30	57
16	Signal transduction	4.79E-16	217	2,786
17	Interferon signaling	2.30E-14	47	197
18	Diseases associated with the TLR signaling cascade	3.40E-14	20	24
19	Diseases of immune system	3.40E-14	20	24
20	Adaptive immune system	9.90E-14	96	745

### Network Construction, Analysis and Iteration

**FIGURE 4:**
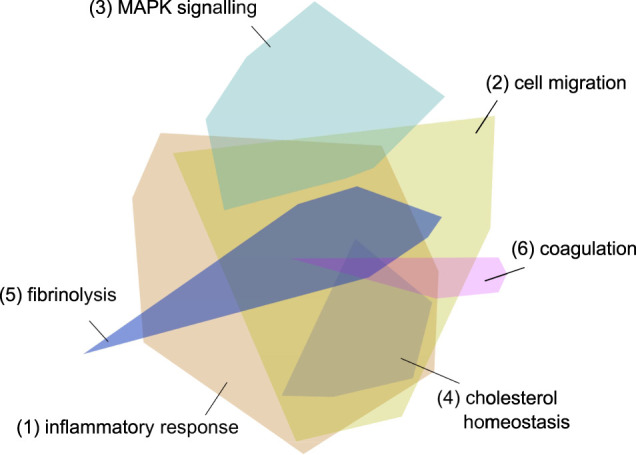
Initial network - the 556 imported genes from the graph. The different colored modules reflect clusters of protein interactions which reflect specific pathways and processes. The modules 1 and 2 reflect inflammatory processes, module 3 signaling pathways, module 4 cholesterol metabolism and modules 5 and 6 coagulation cascades. The three processes most strongly associated with each module are listed in Table 6.

After a qualitative graph evaluation, 556 host proteins were exported from the graph as a result of the graph pattern query “What are the proteins most associated with COVID-19 viral processes?”. The initial network, created by importing the 556 host proteins into the network exploration tool, consisted of 2,286 protein-protein interactions and 16 modules (Figure 4).

Further context was then provided by a literature search for terms relating to viral entry, in which the 500 proteins most commonly co-occurring in the same paragraph with such terms were identified. The top 250 proteins that were found in the initial network, and in the co-occurrence results, yielded a second network (Network 2) that had strong relevance to “viral processes” in the SARS-CoV-2 customised knowledge graph, as well as a strong “viral entry” association from literature. Comparison of this network with the original showed that the top 25 pathways were enriched in specific inflammatory response pathways, (Supplementary Table 1). Similarly, the top 25 biological processes were enriched in viral and inflammation specific processes (Supplementary Table 2). An assessment of the functions of each module (or cluster of protein interactions) revealed a small cluster of proteins containing clathrin, AP2M1 and AAK1, which are associated with endocytosis and membrane trafficking. This suggested that SARS-CoV-2 infection could be mediated by clathrin mediated endocytosis (CME).

In order to include possible drug targets associated with CME, the 10 proteins associated with clathrin in the network were further enriched with the proteins and relationships of the CME pathway. A literature paragraph co-occurrence search for “clathrin-mediated viral endocytosis” was used to identify 250 proteins, 52 of which were already in Network 2. The 198 new proteins were then added, resulting in Network 3, which included clathrin associated pathways and “receptor-mediated endocytosis” in the top 10 enriched biological processes (Supplementary Table 3).

Finally, given the prominent role of cytokine-mediated inflammation in COVID-19 disease, a third iteration was performed to enrich for immune/inflammatory response proteins related to endothelial and epithelial inflammation with cytokine signaling. Adding the top 250 protein co-occurrences from this search increased the network protein count by 209 proteins and added metadata to 41 already existing in the network.

**FIGURE 5:**
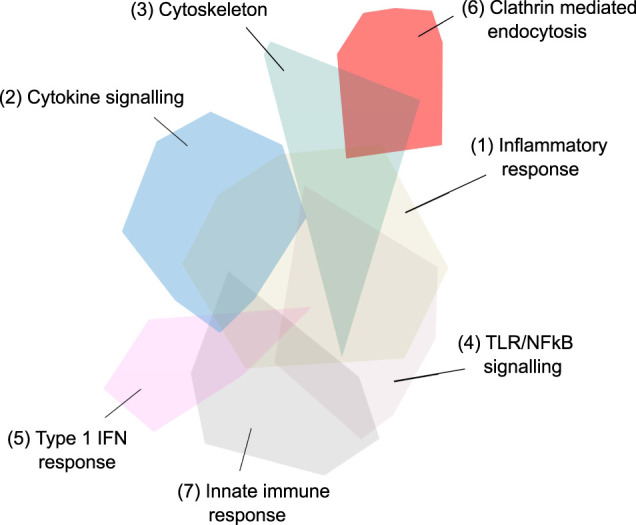
Third iteration, fourth and final network - 657 genes in 20 network modules identified via Louvain-based community detection using protein-protein interactions. The largest 7 modules are annotated.

The top biological processes and pathways of the final network (Network 4) are summarised in Tables 2, 3 and Figure 5.

**TABLE 3:** Top 20 enriched biological processes (Gene Ontology) in the final network.

Rank	Name	*p* value	Matched	Gene set
1	Cytokine-mediated signaling pathway	1.04E-71	116	279
2	Inflammatory response	1.49E-46	100	381
3	Membrane organisation	4.63E-44	66	133
4	Immune response	9.65E-42	85	297
5	Viral entry into host cell	1.83E-36	51	89
6	Innate immune response	2.40E-36	94	457
7	Positive regulation of interleukin-6 production	1.71E-30	45	89
8	Cellular response to lipopolysaccharide	6.86E-29	53	157
9	Defense response to virus	1.04E-28	58	200
10	Positive regulation of NF-kappaB transcription factor activity	7.07E-27	51	160
11	Response to virus	1.67E-25	42	104
12	Positive regulation of interleukin-8 production	1.40E-22	31	54
13	Endocytosis	3.11E-22	48	184
14	Positive regulation of I-kappaB kinase/NF-kappaB signaling	3.78E-22	48	185
15	Signal transduction	2.14E-21	108	1,006
16	Positive regulation of tyrosine phosphorylation of STAT protein	2.92E-21	32	68
17	Type I interferon signaling pathway	8.02E-20	30	65
18	I-kappaB kinase/NF-kappaB signaling	1.12E-19	30	66
19	Chemokine-mediated signaling pathway	1.57E-19	30	67
20	Positive regulation of tumor necrosis factor production	1.78E-19	27	49

The major pathways represented in the final network are summarised in Table 2. Multiple aspects of biology are reflected in this network, summarising protein-protein interactions implicated in virus induced inflammation, host response to the virus, virus and membrane trafficking as well as the major signaling pathways involved in these processes. One module of 31 proteins was highly enriched in proteins associated with clathrin mediated endocytosis and membrane trafficking (AAK1, ADRB2, AP1G1, AP2A1, AP2B1, AP2M1, AP2S1, ARF1, ARF6, ARRB1, ARRB2, ATG16L1, CLTA, CLTB, CLTC, CLTCL1, EPN1, EPN2, EPS15, EPS15L1, FCHO1, FCHO2, FZD5, GAK, ITSN1, ITSN2, NECAP1, NUMB, PICALM, RAB4A, SGIP1). We hypothesised that inhibition of this process would reduce SARS-CoV-2 infection and thus ameliorate the COVID-19 disease.

In summary, the original network contained host protein relationships associated with virus-host interactions including those involved in viral infection of cells and inflammation. These processes were then enriched in order to identify pathways and proteins which could be targeted in order to treat these infections. In total, three iterations of network creation and modification were required to enhance the network (Figure 3) involving 1) the addition of genes to the network, 2) a filtering out of genes from the network, and/or 3) the addition of metadata to genes already existing in the network.

### Identification of Drugs Inhibiting Endocytosis and Inflammation

The modules relevant to CME and cytokine signaling were then examined to see whether there were approved drugs which inhibited them.

**FIGURE 6:**
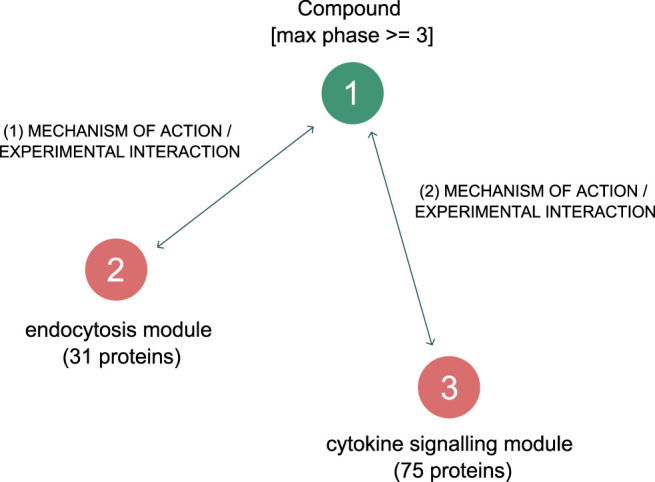
Graph pattern for finding approved drugs that are selective and effective against the two hypothesised driving mechanisms behind SARS-CoV-2 induced COVID-19.

The graph pattern query tool was employed a second time - to create simple graph pattern consisting of three nodes and two relationships is shown in Figure 6. The first node in the pattern is a defined protein group, containing the “endocytosis” related proteins listed above. The second node in the pattern is a defined protein group containing all proteins from the “cytokine signaling” module in the network- a total of 75 proteins. Lastly, the third node in the pattern is an undefined compound, which is constrained to be a clinically approved compound. The two connecting relationships in the pattern are drug interaction relationships, which possess information as to the efficacy of the drug on the protein (usually an IC50 or Kd value).

Executing the pattern returned 16 approved drugs (Table 4) that were reported to inhibit endocytosis proteins, and cytokine response proteins. Of these, drugs 8 to 16 had pKd values too low to inhibit the NAK enzymes at therapeutic exposures.

**TABLE 4:** Approved drugs associated with both endocytosis and cytokine signaling. The drugs approved by the FDA or EMA are indicated with their reported pKd values for AAK1, BMP2K and GAK, and the targets in the cytokine signaling module (Figure 5) which they are reported to inhibit are listed. The highest pChembl value is that reported for the first listed target (i.e. for baricitinib the pChembl value is for JAK2). The drugs are listed in order of affinity for AAK1.

	Drug	AAK1	GAK	BMP2K	Cell based/Cell free	Cmax free (nM)	Cytokine signaling targets	Highest pChembl
		pKd	pKd	pKd				
1	SUNITINIB	7.96	7.7	8.26	1–5	8	[LCK, MAP2K2, JAK2, TYK2, JAK1, PTK2B]	6.6
2	BARICITINIB	7.88	5.33	7.4	2–5	68	[JAK2, JAK1, JAK3, TYK2]	9.1
3	FEDRATINIB	7.46	7.33	7.51	30–100	170	[JAK2, LCK, TYK2, JAK1, JAK3, BTK, PTK2B]	9.0
4	MIDOSTAURIN	6.38	<4.48	6.6	1	<17	[LCK, JAK2, TYK2, SYK, JAK1, JAK3, PTK2B]	6.6
5	RUXOLITINIB	5.45	<4.48	6.68	7	45	[JAK2, JAK1, TYK2, JAK3]	10.4
6	BOSUTINIB	5.32	7.38	6.46	25	18	[MAP2K2, LCK, EPHA2, SYK, BTK, PTK2B]	8.0
7	PALBOCICLIB	5.38	<4.48	—	23	35	[JAK3]	7.2
8	ERLOTINIB	<4.48	6.29	5.51		180	[LCK, JAK3]	6.6
9	DASATINIB	<4.48	6.8	—		7	[LCK]	9.7
10	DACOMITINIB	<4.48	6.77	—		4	[BTK, LCK, EPHA2, TYK2, JAK3, SYK]	6.2
11	AFATINIB	<4.48	5.75	—		8	[LCK]	7.0
12	FOSTAMATINIB	<4.48	<4.48	—		15	[SYK]	7.8
13	PAZOPANIB	<4.48	<4.48	—		<1,000	[LCK]	6.3
14	TRAMETINIB	<4.48	<4.48	—		1	[MAP2K2]	8.8
15	GEFITINIB	<4.48	6.02	—		19	[LCK]	6.4
16	ALPELISIB	<4.48	5.3	—		560	[PIK3CG]	6.6

The free therapeutic plasma concentration and the observed reduction in potency seen in cell-based assays when acting on their primary targets was then taken into account when prioritising these agents. Baricitinib shows a small reduction in potency in cell-based assays (as little as 2-fold depending on the assay, McInnes et al., 2019) and an unbound Cmax indicating that therapeutic exposures would be sufficient to inhibit AAK1 and the related NAK enzyme BIKE or BMP2K. Although sunitinib was amongst the most potent of the approved inhibitors, the unbound Cmax achieved on therapeutic dosing (<10 nM) was deemed insufficient to inhibit the cytokine signaling enzymes given the low pChembl value (Table 4). The reduction in potency from cell-free to cell-based assays for fedratinib was large (Wernig et al., 2008), indicating that it was also unlikely to be an effective NAK inhibitor at therapeutic doses *in vivo*. Using these criteria, it was clear that baricitinib had the pharmacokinetic and pharmacodynamic properties required for repurposing as a combined JAK/NAK inhibitor i.e. a combined anti-viral anti-inflammatory agent. In addition, baricitinib is cleared through the kidneys, suggesting that combination therapy with directly acting and hepatically metabolised antivirals such as remdesivir was a likely possibility (Stebbing et al., 2020a), a hypothesis that was assessed in the ACTT-2 randomised clinical trial (Kalil et al., 2021).

### Clinical Trials of Baricitinib in COVID-19

Multiple observational clinical trials have shown that baricitinib was safe for administration in COVID-19 patients, with reduced mortality rates and/or accelerated recovery from the infection. Table 5 summarises some aspects of these disparate trials. The first (Stebbing et al., 2020b) involved the treatment of 4 patients in Milan, Italy, all of them recovered. This was soon followed by other reports from Italy, Bronte et al. (2020) reported on the treatment of consecutive groups of patients in Verona where baricitinib reduced the mortality by 95%, while Cantini et al. (2020) reported that no patients taking baricitinib died and there was a strong reduction in time to recovery. Even in severely ill and critical patients baricitinib was safe and well tolerated (Titanji et al., 2020). In both Spain and Italy propensity matched cohorts of patients showed baricitinib reduced mortality by between 40 and 60% (Stebbing et al., 2021). The randomised ACTT-2 trial in which baricitinib was administered with remdesivir confirmed its therapeutic efficacy when compared to treatment with remdesivir alone (1,033 patients randomised 1:1). In this study the combination of baricitinib and remdesivir resulted in a statistically significant reduction in hospital stay and a reduction in mortality of 35% in all hospitalised patients, and of approximately 50% in those patients requiring oxygen supplementation on admission. In the same patient group, the time to recovery was shortened from 18 days (remdesivir alone) to 10 days (combination) (Kalil et al., 2021). Partly as a result of this trial and the data from observational trials, the FDA granted an Emergency-Use-Authorisation in November 2020. The CoV-BARRIER trial of baricitinib with standard of care vs standard of care with baricitinib also showed a 38% reduction in hospitalised patient mortality (Marconi et al., 2021), despite the fact that 79% of the patients were also treated with corticosteroids.

**TABLE 5:** Summary of clinical efficacy of baricitinib. Stebbing et al., 2020a, No controls, 4 patients; Bronte et al., 2020, with hydroxychloroquine, 8 mg/day baricitinib for 2 days, followed by 4 mg/day for 7 days, consecutive patients admitted with COVID; Titanji et al., 2020, no controls, 2–4 mg baricitinib 5–7 days with hydroxy-chloroquine, 15 moderate-critical patients; Cantini et al., 2020, moderate patients with lopinavir/ritonavir, 88% of those on baricitinib recovered after 14 days (control 14%); Stebbing et al., 2021 propensity matched patients, with antiviral medications including lopinavir/ritonavir; Kalil et al., 2021: Patients requiring high flow oxygen or non-invasive ventilation (ordinal groups 5 and 6), mortality at day 28, with remdesivir, median time to recovery 10 days (control 18 days). Marconi et al., 2021: 38% reduction in mortality at day 28, with standard of care. IMV: invasive mechanical ventilation.

	Mortality/IMV n (%)		Patient numbers	
Observational studies	Control	Baricitinib	Control	Baricitinib
Stebbing et al. (2020b)	—	0	—	4
Bronte et al., 2020	25	1	20	20
Titanji et al. (2020)	—	4	—	15
Cantini et al., 2020	7	0	78	78
Propensity matched trials				
Stebbing et al. (2021)	13	5	37	37
Stebbing et al. (2021)	16	9	46	46
Randomised trial				
Kalil et al. (2021)	25	12	356	341
Marconi et al. (2021)	100	62	761	764

**TABLE 6:** Biological process enrichment for the six largest modules in the initial network.

Module	Process rank	Process name	Adjusted *p* value	Matched in network	Total gene set
1	1	Inflammatory response	3.50E-33	54	381
1	2	Cytokine-mediated signaling pathway	3.14E-29	45	279
1	3	Immune response	5.04E-19	36	297
2	1	Positive regulation of cell migration	2.48E-05	10	227
2	2	Leukocyte migration	3.46E-05	8	130
2	3	Receptor internalisation	3.46E-05	6	42
3	1	Positive regulation of protein kinase B signaling	2.05E-34	26	166
3	2	Positive regulation of cell population proliferation	1.69E-26	28	497
3	3	MAPK cascade	7.44E-25	23	262
4	1	Cholesterol homeostasis	3.24E-12	8	84
4	2	Chylomicron remnant clearance	5.44E-10	5	8
4	3	Cholesterol metabolic process	2.72E-08	6	75
5	1	Fibrinolysis	8.01E-06	4	20
5	2	Positive regulation of fibrinolysis	0.02912269	2	4
6	1	Blood coagulation	1.00E-10	8	173
6	2	Blood coagulation, intrinsic pathway	2.26E-06	4	17
6	3	Platelet activation	1.20E-03	4	102

## Discussion

We have described here how we enriched our biomedical knowledge graph (Paliwal et al., 2020) using NLP, using it to identify host biological processes and pathways that are impacted by SARS-CoV-2 infection. Through workflow iterations of graph pattern querying and protein-protein interaction network exploration, a final network was found to be significantly enriched for multiple disease mechanisms of interest, in particular - viral infection and cytokine-mediated inflammation, two processes involved in COVID-19. Finally, we identified approved drugs capable of inhibiting these processes and, after consideration of dosage and pharmacokinetics, we selected baricitinib as an inhibitor of both viral entry (through inhibition of AAK1 and BMP2K and so CME) as well as cytokine mediated inflammation through JAK1/2 inhibition.

A strength of this approach lies in the human-machine interaction which enabled scientists to identify relevant biological contexts through interactive and visual presentations of data, combined with tooling that enabled them to augment system-derived biological representations with their own knowledge. This augmentation guided the workflow in productive directions within hourly timeframes. Thus, we had enriched our initial observations of viral process-associated targets with clathrin-mediated endocytosis pathways, while also enriching for the required anti-inflammatory properties of the repurposed drug. The result of these iterations led scientists toward searching for appropriate drugs which could inhibit both the virus and the inflammation associated with COVID-19.

This work was carried out in January 2020 and the recommendation to use this drug was published in February (Richardson et al., 2020). Partly as a result of these predictions multiple observational trials ensued (Table 5), all showing that baricitinib was safe to be administered to COVID-19 patients and reducing mortality rates or accelerating recovery from the virus. These results were consistent with the predicted antiviral and anti-inflammatory action of baricitinib. The predicted antiviral efficacy of baricitinib has also been demonstrated in human liver spheroids and organoids (Stebbing et al., 2020b; Stebbing et al., 2021) while the anti-inflammatory effects of this drug are well known and underpin its approved use in rheumatoid arthritis.

The therapeutic efficacy of baricitinib in COVID-19 was finally proven in the randomised ACTT-II trial in which baricitinib in combination with remdesivir reduced time to recovery and hospitalised patient mortality (Kalil et al., 2021). Partly as a result of the ACTT-II trial and the data from observational trials, the FDA granted an EUA in November 2020. Since then Eli Lilly and Co have reported a 38% reduction in mortality with baricitinib in the CoV BARRIER trial, despite the fact that 79% of the patients were taking corticosteroids. In contrast NIAID have terminated (due to futility) the ACTT-IV trial comparing baricitinib to dexamethasone, both in the presence of remdesivir. This suggests that, in the presence of the anti-viral remdesivir, corticosteroids and baricitinib have equivalent beneficial effects, but in its absence baricitinib improves the response to standard of care including corticosteroids. It is tempting to speculate that this is due to the intrinsic anti-viral effect of baricitinib identified in this report. One other Phase 3 trial testing baricitinib is currently underway (NCT04390464, TACTIC-R), which is due to report later in 2021.

The selection of baricitinib as the best possible approved drug for inhibiting both viral entry and cytokine signaling was based mainly on whether the free exposure of the drug on therapeutic dosing could be sufficient to inhibit both processes. Due to the lack of cell-based assay data for the NAK enzymes we estimated the likely potency of the drugs in cell assays using the reduction in drug potency seen at their primary targets. Although this method is not very accurate it gave an estimation of drug potency *in vivo* which served to prioritise the drugs in the light of the approaching pandemic.

The pKd values of sunitinib for the JAKs were seen to be too low for it to have a significant effect on cytokine signaling. The very high protein binding of midostaurin and pazopanib makes it difficult to estimate their likely therapeutic exposures, but the pKd values for the NAK enzymes suggested these compounds were unlikely to inhibit CME. Similarly, the low pKd values of the remaining drugs in Table 4 for the NAK enzymes, especially when considered in the light of their therapeutic exposures, suggested that they also would be unlikely to inhibit CME.

It is perhaps no surprise that the antiviral targets of baricitinib (AAK1, BMP2K and GAK) are kinases, particularly as ATP competitive kinase inhibitors are notoriously unselective. There was however a potential drawback to using a JAK1/2 inhibitor such as baricitinib for the treatment of viral diseases in that the Type I IFN antiviral response also utilises JAK/STAT signaling pathways. Therefore, it is possible that blockade of this response could worsen the disease by weakening the antiviral IFN-mediated response. There were reasons to doubt that this would be a significant effect since IFN therapy has had inconsistent effects in antiviral trials (Channappanavar et al., 2019), and in the SARS epidemic patients with severe disease who were discharged from hospital had low levels of Type 1 IFN stimulated gene expression, whereas those who died had prominent Type 1 IFN activity (Cameron et al., 2007). Although the hyper-inflammatory severe phase of the COVID-19 illness occurs when the viral load is already decreasing, viral shedding can occur up to 6 weeks after disease onset in patients experiencing severe disease, (Chen et al., 2020; Wang et al., 2020), showing that antiviral treatment is still required during the later phases of the illness.

There are some important aspects to this KG and the tools used to query it which enabled the identification of baricitinib in 48 h. These include the rapid NLP enrichment of a knowledge graph, rapid feedback loops of questioning, answering and workflow iteration, flexible and malleable starting points (biological processes, diseases, pathways, phrases and gene set representations of these) and the ability to rapidly contextualise biological networks through literature mining. The successful application of this combination of machine and human drug repurposing in the pandemic suggests that the same approach could be used to find treatments for other diseases, including those which are rare or neglected.

Accordingly, our investigation included considerable guidance of machine enriched data by human intuition and knowledge. A lack of high-quality data, and gaps in the available data, can limit the capabilities of machine trained systems. These limitations can be compensated for by pragmatic approaches centered around human guidance. The combined HCI-KDD method of a machine, capable of reading a repository of knowledge at scale, directed by an expert is, in theory, a powerful partnership, provided certain capabilities are in-place–such as building the system to be flexible enough to allow the user to repeatedly append and amend the content throughout workflow stages. For example, in this paper we describe the means by which a knowledge graph was enhanced, leading to increased confidence in both the general biological mechanisms and specific targets of interest.

It is worth noting that when multiple iterations are made, trends and patterns detected in the data can also come and go in rapid succession–and one important pattern detection method used in this research was the topological community detection in networks. When iterating on multiple networks–the communities identified can drastically change size and location based on the addition or omission of a single network node, which poses a challenge when interpreting them across multiple iterations of the same network. In this study, we observed the endocytosis module, an immune system module, and an inflammation module, were all often of the same size in relation to each other and maintained similar inter-module relationships and location in the network. This suggested the importance, and prevalence of those three mechanistic areas across the network iterations, so the fragility of dynamic topological community detection became instead a signal for detecting robust biology across workflow iterations.

Although automated methods are largely free of user bias, the biomedical literature suffers from different types and levels of biological and informational bias. In the tools used in this workflow, several metrics were embedded in views while interpreting results; examples of these are normalised point-wise mutual information scores for measuring the specificity of literature co-occurrences, and Jaccard similarity scores for measuring knowledge graph entity similarity. In addition, the tools also feature visual and interaction techniques that provide the expert with multiple options of assessing relevancy (i.e. module coloring and topological clustering rather than informational clustering) and changing visual encodings of information at hand. There is always a risk in a collaborative human-machine knowledge discovery approach, in that the inherent conscious or unconscious bias of the user will guide the workflow toward well understood areas. Mitigation of this risk lies both with the user and the design of the system, the success of which is seen in experimental validation of predictions.

Overall, the methodology described here points to a pragmatic balance between scientist expertise and machine power, in the face of an urgent worldwide health emergency. While not instant, or push-button, this paper demonstrates that it is possible to rapidly interrogate huge volumes of data to purposeful effect, with clinically confirmed outcomes. While this approach generated one of the earliest data-driven discoveries in response to the COVID-19 pandemic, it does not reflect BenevolentAI’s typical approach to de-novo drug discovery, but we do anticipate that it will be increasingly used by many in the future.

## Data Availability

The original contributions presented in the study are publicly available. The data analysed in this study can be accessed from the following sources: KEGG https://www.genome.jp/kegg/ Reactome https://reactome.org/ StringDB https://string-db.org/ SiGNoR https://signor.uniroma2.it/ BioGRID https://thebiogrid.org/ ChEMBL https://www.ebi.ac.uk/chembl/ CTD http://ctdbase.org/ GWAS https://www.ebi.ac.uk/gwas/ Gene Ontology http://geneontology.org/ OMIM https://www.omim.org/ Mondo https://mondo.monarchinitiative.org/ Further inquiries can be directed to the corresponding author.
