# Supplementary material for: Expert-Augmented Computational Drug Repurposing Identified Baricitinib as a Treatment for COVID-19
Source: Front Pharmacol. 2021 Jul 28;12:709856. doi: 10.3389/fphar.2021.709856 (PMC8356560; doi:10.3389/fphar.2021.709856)
Supplement: Supplementary file 1 [file DataSheet1.docx]

**Table S1** First iteration (adding ‘viral entry’ context) - Changes in top 25 enriched pathways

| **Pathway name** | **Change** | **Change in matched genes** |
| --- | --- | --- |
| Interferon alpha/beta signaling | New |  |
| Interferon Signaling | New |  |
| TRAF3-dependent IRF activation pathway | New |  |
| EPH-Ephrin signaling | New |  |
| Chemokine receptors bind chemokines | New |  |
| TNFs bind their physiological receptors | New |  |
| Antiviral mechanism by IFN-stimulated genes | New |  |
| TRAF6 mediated IRF7 activation | New |  |
| Trafficking and processing of endosomal TLR | New |  |
| ISG15 antiviral mechanism | New |  |
| Cell-Cell communication | New |  |
| DDX58/IFIH1-mediated induction of interferon-alpha/beta | New |  |
| Cell-cell junction organization | New |  |
| TNFR2 non-canonical NF-kB pathway | New |  |
| EPHB-mediated forward signaling | New |  |
| Cell junction organization | New |  |
| Interleukin-10 signaling | New |  |
| Toll-like Receptor Cascades | New |  |
| Negative regulators of DDX58/IFIH1 signaling | New |  |
| Cell surface interactions at the vascular wall | New |  |
| Nectin/Necl  trans heterodimerization | New |  |
| Cytokine Signaling in Immune system | Promoted | -46 |
| Innate Immune System | Promoted | -46 |
| Signaling by Interleukins | Demoted | -40 |
| Immune System | - | -94 |
| Heparan sulfate/heparin (HS-GAG) metabolism | Removed |  |
| Extracellular matrix organization | Removed |  |
| Glycosaminoglycan metabolism | Removed |  |
| HS-GAG biosynthesis | Removed |  |
| Signaling by Receptor Tyrosine Kinases | Removed |  |
| Constitutive Signaling by Aberrant PI3K in Cancer | Removed |  |
| Interleukin-4 and Interleukin-13 signaling | Removed |  |
| Disease | Removed |  |
| Hemostasis | Removed |  |
| PI5P,PP2A and IER3 Regulate PI3K/AKT Signaling | Removed |  |
| PI3K/AKT Signaling in Cancer | Removed |  |
| Response to elevated platelet cytosolic Ca2+ | Removed |  |
| Signal Transduction | Removed |  |
| Negative regulation of the PI3K/AKT network | Removed |  |
| HS-GAG degradation | Removed |  |
| Platelet degranulation | Removed |  |
| Regulation of Insulin-like Growth Factor (IGF) transport... | Removed |  |
| Neutrophil degranulation | Removed |  |
| Degradation of the extracellular matrix | Removed |  |
| Platelet activation,signaling and aggregation | Removed |  |
| A tetrasaccharide linker sequence is required for GAG synthesis | Removed |  |

**Table S2** Second iteration (adding ‘clathrin mediated endocytosis’ context) - Changes in top 25 enriched processes

| **Process name** | **Change** | **Changed in matched genes** |
| --- | --- | --- |
| defense response to virus | New |  |
| response to virus | New |  |
| type I interferon signaling pathway | New |  |
| negative regulation of viral genome replication | New |  |
| innate immune response | New |  |
| positive regulation of interferon-beta production | New |  |
| viral process | New |  |
| positive regulation of interferon-alpha production | New |  |
| T cell costimulation | New |  |
| receptor internalization | New |  |
| cellular response to exogenous dsRNA | New |  |
| chemokine-mediated signaling pathway | New |  |
| tumor necrosis factor-mediated signaling pathway | New |  |
| fusion of virus membrane with host plasma membrane | New |  |
| ephrin receptor signaling pathway | New |  |
| response to interferon-beta | New |  |
| response to interferon-alpha | New |  |
| positive regulation of interleukin-6 production | New |  |
| leukocyte migration | New |  |
| inflammatory response | New |  |
| cytokine-mediated signaling pathway | New |  |
| adaptive immune response | New |  |
| immune response | Promoted | +32 |
| viral entry into host cell | Promoted | +15 |
| cell adhesion | - | 0 |
| leukocyte migration | Demoted | -18 |
| immune response | Demoted | -24 |
| cell adhesion | Demoted | -32 |
| cytokine-mediated signaling pathway | Demoted | -41 |
| inflammatory response | Demoted | -45 |
| positive regulation of protein kinase B signaling | Removed |  |
| positive regulation of gene expression | Removed |  |
| positive regulation of ERK1 and ERK2 cascade | Removed |  |
| positive regulation of cell population proliferation | Removed |  |
| glycosaminoglycan biosynthetic process | Removed |  |
| cellular protein metabolic process | Removed |  |
| extracellular matrix organization | Removed |  |
| positive regulation of protein phosphorylation | Removed |  |
| signal transduction | Removed |  |
| neutrophil degranulation | Removed |  |
| positive regulation of cell migration | Removed |  |
| positive regulation of peptidyl-tyrosine phosphorylation | Removed |  |
| glycosaminoglycan catabolic process | Removed |  |
| platelet degranulation | Removed |  |
| MAPK cascade | Removed |  |
| angiogenesis | Removed |  |
| positive chemotaxis | Removed |  |
| negative regulation of apoptotic process | Removed |  |
| cellular response to lipopolysaccharide | Removed |  |

**Table S3** Second iteration (adding ‘clathrin-mediated endocytosis’) - Changes in top 25 enriched pathways

| **Pathway name** | **Change** | **Change in matched genes** |
| --- | --- | --- |
| Clathrin-mediated endocytosis | New |  |
| Vesicle-mediated transport | New |  |
| Membrane Trafficking | New |  |
| Cargo recognition for clathrin-mediated endocytosis | New |  |
| trans-Golgi Network Vesicle Budding | New |  |
| Golgi Associated Vesicle Biogenesis | New |  |
| EPH-ephrin mediated repulsion of cells | New |  |
| Signaling by Receptor Tyrosine Kinases | New |  |
| Retrograde neurotrophin signalling | New |  |
| Lysosome Vesicle Biogenesis | New |  |
| Nervous system development | New |  |
| Axon guidance | New |  |
| Plasma lipoprotein clearance | New |  |
| Signaling by EGFR | New |  |
| Signaling by NTRKs | New |  |
| Gap junction degradation | New |  |
| EGFR downregulation | New |  |
| Immune System | Demoted | +50 |
| Cytokine Signaling in Immune system | Demoted | +19 |
| EPH-Ephrin signaling | Demoted | +12 |
| Innate Immune System | Demoted | +25 |
| Interferon Signaling | Demoted | +1 |
| EPHB-mediated forward signaling | Demoted | +5 |
| Cell-Cell communication | Demoted | +7 |
| Interferon alpha/beta signaling | - | 0 |
| TRAF3-dependent IRF activation pathway | Removed |  |
| Chemokine receptors bind chemokines | Removed |  |
| TNFs bind their physiological receptors | Removed |  |
| Antiviral mechanism by IFN-stimulated genes | Removed |  |
| TRAF6 mediated IRF7 activation | Removed |  |
| Trafficking and processing of endosomal TLR | Removed |  |
| ISG15 antiviral mechanism | Removed |  |
| DDX58/IFIH1-mediated induction of interferon-alpha/beta | Removed |  |
| Cell-cell junction organization | Removed |  |
| TNFR2 non-canonical NF-kB pathway | Removed |  |
| Signaling by Interleukins | Removed |  |
| Cell junction organization | Removed |  |
| Interleukin-10 signaling | Removed |  |
| Toll-like Receptor Cascades | Removed |  |
| Negative regulators of DDX58/IFIH1 signaling | Removed |  |
| Cell surface interactions at the vascular wall | Removed |  |
| Nectin/Necl  trans heterodimerization | Removed |  |
